# Phase‐shifted transverse relaxation orientation dependences in human brain white matter

**DOI:** 10.1002/nbm.4925

**Published:** 2023-04-02

**Authors:** Yuxi Pang

**Affiliations:** ^1^ Department of Radiology University of Michigan Ann Arbor Michigan USA

**Keywords:** anisotropic diffusivity direction, magic angle effect, magnetic susceptibility anisotropy, phase shift, principal diffusivity direction, transverse relaxation orientation dependence, white matter

## Abstract

This work aimed to demonstrate an essential phase shift $\varepsilon_{0}$ for better quantifying $R_{2}$ and $R_{2}^{*}$ in human brain white matter (WM), and to further elucidate its origin related to the directional diffusivities from standard diffusion tensor imaging (DTI). $\varepsilon_{0}$ was integrated into a proposed generalized transverse relaxation model for characterizing previously published $R_{2}$ and $R_{2}^{*}$ orientation dependence profiles in brain WM, and then comparisons were made with those without $\varepsilon_{0}$. It was theorized that anisotropic diffusivity direction ε was collinear with an axon fiber subject to all eigenvalues and eigenvectors from an apparent diffusion tensor. To corroborate the origin of $\varepsilon_{0}$, $R_{2}$ orientation dependences referenced by ε were compared with those referenced by the standard principal diffusivity direction Φ at *b*‐values of 1000 and 2500 (s/mm^2^). These $R_{2}$ orientation dependences were obtained from $T_{2}$‐weighted images (*b =* 0) of ultrahigh‐resolution Connectome DTI datasets in the public domain. A normalized root‐mean‐square error ( NRMSE%) and an F‐test were used for evaluating curve‐fittings, and statistical significance was considered to be a *p* of 0.05 or less. A phase‐shifted model resulted in significantly reduced NRMSE% compared with that without $\varepsilon_{0}$ in quantifying various $R_{2}$ and $R_{2}^{*}$ profiles, both in vivo and ex vivo at multiple $B_{0}$ fields. The $R_{2}$ profiles based on Φ manifested a right‐shifted phase ( $\varepsilon_{0}>0$) at two *b*‐values, while those based on ε became free from $\varepsilon_{0}$. For all phase‐shifted $R_{2}$ and $R_{2}^{*}$ profiles, $\varepsilon_{0}$ generally depended on the directional diffusivities by $\tan^{-1}\left(D_{⊥}/D_{∥}\right)$, as predicted. In summary, a ubiquitous phase shift $\varepsilon_{0}$ has been demonstrated as a prerequisite for better quantifying transverse relaxation orientation dependences in human brain WM. Furthermore, the origin of $\varepsilon_{0}$ associated with the directional diffusivities from DTI has been elucidated. These findings could have a significant impact on interpretations of prior $R_{2}$ and $R_{2}^{*}$ datasets and on future research.

## Graphical abstract

A ubiquitous phase shift has been demonstrated as essential for better quantifying $R_{2}$ and $R_{2}^{*}$ orientation dependences in human brain white matter. The origin of this phase shift has been further elucidated as quantitatively linked to the directional diffusivities from standard diffusion tensor imaging. The outcome from this work could have a significant impact on future research and on interpretations of previously published $R_{2}$ and $R_{2}^{*}$ datasets in human brain white matter from both adults and newborns.

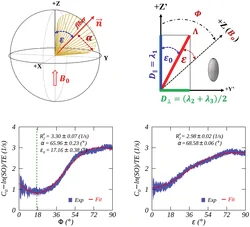

**Abbreviations**
ADD: anisotropic diffusivity direction
CST: corticospinal tract
FA: fractional anisotropy
HCFM: hollow cylinder fiber model
LRF: laboratory reference frame
MAE: magic angle effect
MO: mode of anisotropy
MTS: magnetization transfer saturation
MWF: myelin water fraction
NRMSE: normalized root‐mean‐square error
PAS: principal axis system
PDD: principal diffusivity direction
RDC: residual dipolar coupling
ROI: region of interest
TPF: transverse pontine fibers
WM: white matter

## 1 INTRODUCTION

In recent years, transverse relaxation ( $R_{2}$ and $R_{2}^{*}$) orientation dependences in brain white matter (WM) have been extensively studied both in vivo^1^, ^2^, ^3^, ^4^, ^5^, ^6^, ^7^, ^8^, ^9^ and ex vivo^10^, ^11^, ^12^, ^13^, ^14^ at multiple magnetic $B_{0}$ fields. These enduring efforts are motivated by the ultimate goal of specifically quantifying microstructural alterations in myelination secondary to different pathological conditions (e.g., multiple sclerosis^15^, ^16^) or at different stages of brain development (e.g., infant brain^4^, ^17^, ^18^). Based on the knowledge of highly organized axonal microstructures and associated magnetic susceptibility, as well as its orientational anisotropy, several models have been proposed to date.^10^, ^12^, ^13^, ^19^ These models were, however, unable to satisfactorily characterize most of the previously reported $R_{2}$ and $R_{2}^{*}$ orientation dependences, and thus compromised the intrinsic specificity associated with anisotropic $R_{2}$ and $R_{2}^{*}$ in brain WM.

For biological tissues featuring cylindrical shapes such as an axon fiber, $R_{2}^{*}$ from gradient echo sequences is reportedly proportional to sin2ε in the so‐called static dephasing regime, where diffusion phenomena may be disregarded.^20^ Here, the angle ε refers to an axon fiber direction relative to the static magnetic field $B_{0}$ direction. Based on the magnetic susceptibility differences between cylindrical microstructures and magnetic local environments, one of the prior model functions was proposed, including an extra term sin4ε to account for susceptibility anisotropy.^12^ This developed model function was later recast into $R_{2}^{*}=a_{0}+a_{1}\cos2\varepsilon +a_{2}\cos4\varepsilon$.^10^, ^21^

Primarily relying on the so‐called hollow cylinder fiber model (HCFM),^13^ a seemingly different but mathematically equivalent model function has been proposed^1^, ^2^ (i.e., $R_{2}^{*}=b_{0}+b_{1}\sin^{2}\varepsilon +b_{2}\sin^{4}\varepsilon$). More accurately, this HCFM can only analytically predict the term $\sin^{4}\varepsilon$ in the dynamic dephasing regime without considering the signal contribution from myelin water. To minimize the difference between theoretical prediction and measured $R_{2}^{*}$, the term $\sin^{2}\varepsilon$ was heuristically included to account for the influence from myelin water. Interestingly, this specific model function was also suggested for $R_{2}$ orientation dependences based on spin echo sequences.^19^ In this case, an identical model function was derived from diffusion‐mediated decoherence because of magnetic susceptibility alterations at the mesoscopic scale. As $R_{2}^{*}$ is composed of an intrinsic $R_{2}$ part and an external $R_{2}^{\prime }$ contribution from local magnetic field inhomogeneities,^12^ the origin of $R_{2}^{*}$ orientation dependence would be intrinsic in nature if the measured $R_{2}^{*}$ and $R_{2}$ orientational anisotropies are comparable.

The coefficients of those prior model functions may differ depending on what model was utilized when quantifying $R_{2}$ and $R_{2}^{*}$ orientation dependences, rendering ambiguous interpretations of these coefficients. Frequently, these coefficients can become negative numbers,^2^ obviously with no biophysically meaningful basis. More importantly, these models cannot adequately explain some extreme datapoints that have manifested in most of the $R_{2}$ and $R_{2}^{*}$ profiles in the literature.^2^, ^3^, ^22^ Specifically, these extreme datapoints appeared at the measured fiber orientations of Φ≈ 20 °, where $R_{2}$ or $R_{2}^{*}$ became minimum, or at Φ≈80°, where the maximum was found. Here, the measured fiber orientation Φ, like an actual fiber orientation ε, was also referenced to the $B_{0}$ direction.

Although predominantly relying on the susceptibility effect, those previously developed models did not rule out the magic angle effect (MAE) as a potential relaxation mechanism.^1^, ^10^ The MAE has been known for more than half a century,^23^ and it has been described as a remarkable phenomenon for $T_{2}$ (i.e., 1/ $R_{2}$) relaxation in highly organized biological tissues.^24^, ^25^, ^26^ Briefly, the MAE arises from nonaveraging proton residual dipolar coupling (RDC) in heterogenous microenvironments, and it depends on the direction of dipolar interactions relative to $B_{0}$. To date, most of the basic research and clinical applications on the topic have focused on collagen‐rich tissues such as tendon and cartilage.^24^ However, for the human nervous system, the MAE remains elusive, particularly in the brains of adults, even although it has been reported in the peripheral nervous system^27^, ^28^ and recently in the brains of newborns.^17^ When the standard MAE function is appropriately evaluated in a cylindrical system, such as a concentric distribution of water proton RDC around an axon fiber, a generalized MAE function can be formulated.^29^, ^30^ This generalized function can be recast into one of the previous model functions that originated from the susceptibility effect.^21^, ^23^ This finding implies that the underlying relaxation mechanisms cannot be disentangled simply based on different function forms. However, these two relaxation mechanisms can be, in principle, separated according to $R_{2}$ and $R_{2}^{*}$ orientation dependences at different $B_{0}$ fields. This is because the relaxation mechanisms associated with susceptibility depend quadratically on $B_{0}$, while the MAE is independent of $B_{0}$.^31^, ^32^, ^33^

An axon fiber orientation in vivo is predominantly determined by the principal diffusivity direction (PDD) from standard diffusion tensor imaging (DTI).^8^, ^9^ An imaging voxel size is of the order of millimeters in clinical DTI studies, while an average axon fiber diameter is of the order of micrometers in brain WM.^34^ Thus, there will be tens of millions of axon fibers within an imaging voxel, and these axon fibers may not align uniformly along one direction. For instance, different fiber orientation dispersions have been documented in the corpus callosum with highly compact fiber bundles.^35^ Therefore, it remains uncertain whether the PDD can still appropriately represent an axon fiber orientation at the cellular level.

Consistent evidence from previous susceptibility tensor imaging studies has revealed that the principal susceptibility direction significantly deviates from the PDD in brain WM.^36^, ^37^, ^38^ In the literature, susceptibility anisotropy has been attributed to a concentric distribution of lipid molecules within bilayers around an axon fiber.^39^ Similarly, the origin of the MAE has been associated with a concentric distribution of ordered water molecules near the surface of bilayers.^29^, ^40^ Accordingly, the observed $R_{2}$ or $R_{2}^{*}$ orientation dependence profile, if guided by the PDD, ought to be phase shifted.^37^ Regardless of the origin of the underlying relaxation mechanisms, the measured $R_{2}$ and $R_{2}^{*}$ orientation dependences must be described as accurately as possible by any competing model. Hence, this work aimed to demonstrate that a phase shift is a prerequisite for better quantifying $R_{2}$ and $R_{2}^{*}$ orientation dependences in brain WM, and to further elucidate the origin of the phase shift associated with directional diffusivities from DTI.

## 2 THEORY

### 2.1 A unified model for $R_{2}^{*}$ and $R_{2}$ orientation dependences

Previous models for quantifying $R_{2}^{*}$ and $R_{2}$ may be generalized using Equation (1),^10^, ^13^, ^17^, ^19^ where the orientation independent and dependent contributions to the measured $R_{2}^{\exp}$ are, respectively, denoted by $R_{2}^{i}$ and $R_{2}^{a}\;f\left(\alpha ,\varepsilon \right)$.

(1) $$R_{2}^{\exp}=R_{2}^{i}+R_{2}^{a}*f\left(\alpha ,\varepsilon \right)$$

**FIGURE 1:**
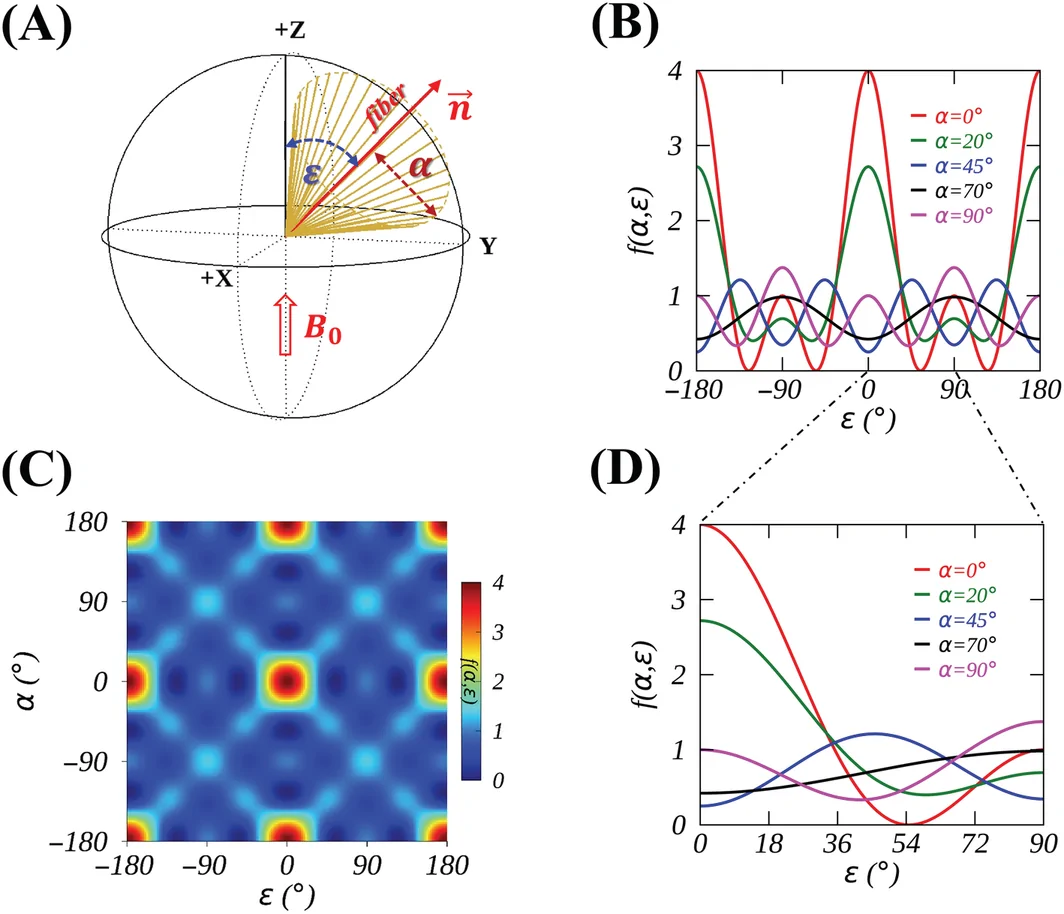
An axially symmetric model for transverse relaxation orientation dependence in brain white matter. (A) A concentric distribution of proton–proton residual dipolar interactions around an axon fiber, which makes an angle α to the proton–proton direction and an angle ε to the static magnetic field $B_{0}$ direction. The normalized orientation dependence function $f\left(\alpha ,\varepsilon \right)$ is profiled for different α angles covering an (B) Extended or a (D) Limited orientation ε range. (C) A detailed orientation dependence of $f\left(\alpha ,\varepsilon \right)$ is given as a 2D map.

In the past, $R_{2}^{a}$ has been substituted by the myelin water fraction (MWF)^16^, ^21^ or magnetization transfer saturation (MTS)^41^ in the case of $R_{2}^{*}$ studies, while it simply refers to the strength of RDC in the case of $R_{2}$.^29^ $f\left(\alpha ,\varepsilon \right)$ is a normalized orientation dependence function for an axially symmetric or cone‐shaped RDC distribution surrounding an axon fiber, as depicted in Figure 1A. This function could be parameterized either by a factored form,^30^ as expressed in Equation (2), or by an expanded form in Equation (3), as originally presented by Berendsen.^23^ Interestingly, this expanded form has been previously used for characterizing transverse relaxation orientation dependences based on magnetic susceptibility anisotropy, rather than the MAE. The reason to provide both function forms herein is to contrast two drastically different relaxation mechanisms, but both parametrized by the same $f\left(\alpha ,\varepsilon \right)$.

(2) $$f\left(\alpha ,\varepsilon \right)=\frac{1}{4}{\left(3\cos^{2}\alpha -1\right)}^{2}{\left(3\cos^{2}\varepsilon -1\right)}^{2}+\frac{9}{8}\left(\sin^{4}\alpha \;\sin^{4}\varepsilon +\sin^{2}2\alpha \;\sin^{2}2\varepsilon \right)$$

(3) $$f\left(\alpha ,\varepsilon \right)=A_{0}+A_{1}\cos2\varepsilon +A_{2}\cos4\varepsilon$$

The three interdependent coefficients are defined as follows: $A_{0}=\left(81\cos4\alpha +156\cos2\alpha +467\right)/512$, $A_{1}=\left(180\cos4\alpha +432\cos2\alpha +156\right)/512$, and $A_{2}=\left(315\cos4\alpha +180\cos2\alpha +81\right)/512$. ε is the angle between an axon fiber direction and the $B_{0}$ direction, and α is an opening angle of the cone.

If α becomes zero, $f\left(\alpha ,\varepsilon \right)$ will be shrunk into the familiar term ${\left(3\cos^{2}\varepsilon -1\right)}^{2}$ that is commonly associated with the standard MAE in the literature.^9^, ^10^ The maximum and the minimum $f\left(\alpha ,\varepsilon \right)$ values are $f\left(0{}^{\circ},0{}^{\circ}\right)=4$ and $f\left(0{}^{\circ},54.7{}^{\circ}\right)=0$, respectively. The multiple symmetries of $f\left(\alpha ,\varepsilon \right)$ intrinsic to an axially symmetric system are easily recognized in Equation (2), for example, $f\left(\alpha ,\varepsilon \right)=$ $f\left(\varepsilon ,\alpha \right)$ $=f\left(\alpha ,\varepsilon \pm 180{}^{\circ}\right)$. If ε can be accurately determined, $R_{2}^{\exp}$ would be fully quantified by three model parameters, that is, $R_{2}^{i}$, $R_{2}^{a}$, and α, if $f\left(\alpha ,\varepsilon \right)$ is in the factored form, or by $C_{0}=R_{2}^{i}+R_{2}^{a}A_{0}$, $C_{1}=R_{2}^{a}A_{1}$, and $C_{2}=R_{2}^{a}A_{2}$ if $f\left(\alpha ,\varepsilon \right)$ is in the expanded form.

### 2.2 A phase‐shifted $f\left(\alpha ,\varepsilon \right)$ for ex vivo studies

When a brain WM specimen is manually rotated within an MR scanner,^11^, ^12^ an angle Φ between the long axis of the specimen and the $B_{0}$ direction can be determined using an angle‐measuring tool. In a series of measurements on the specimen with different rotations, the first may not necessarily be performed with the long axis collinear with the $B_{0}$ direction. Consequently, the measured $R_{2}^{*}$ or $R_{2}$ profile from a compact axon fiber bundle within the specimen will be out of phase relative to the theoretical $f\left(\alpha ,\varepsilon \right)$ profile, provided that those fibers are aligned with the long axis. Even though the first measurement could start exactly with Φ=0° for one axon fiber tract, there may exist some noncollinear fiber bundles in the same specimen. Therefore, a phase‐shifted $R_{2}^{*}$ or $R_{2}$ orientation dependence profile, that is, $f\left(\alpha ,\Phi -\varepsilon_{0}\right)$, is not unusual in brain WM ex vivo, with either a right shift ( $\varepsilon_{0}>0$) or a left shift ( $\varepsilon_{0}<0$). In other words, the measured $R_{2}^{*}$ or $R_{2}$ profile will become as the theory predicts once the measured profile is shifted by $\varepsilon_{0}$.

### 2.3 A phase‐shifted $f\left(\alpha ,\varepsilon \right)$ for in vivo studies

**FIGURE 2:**
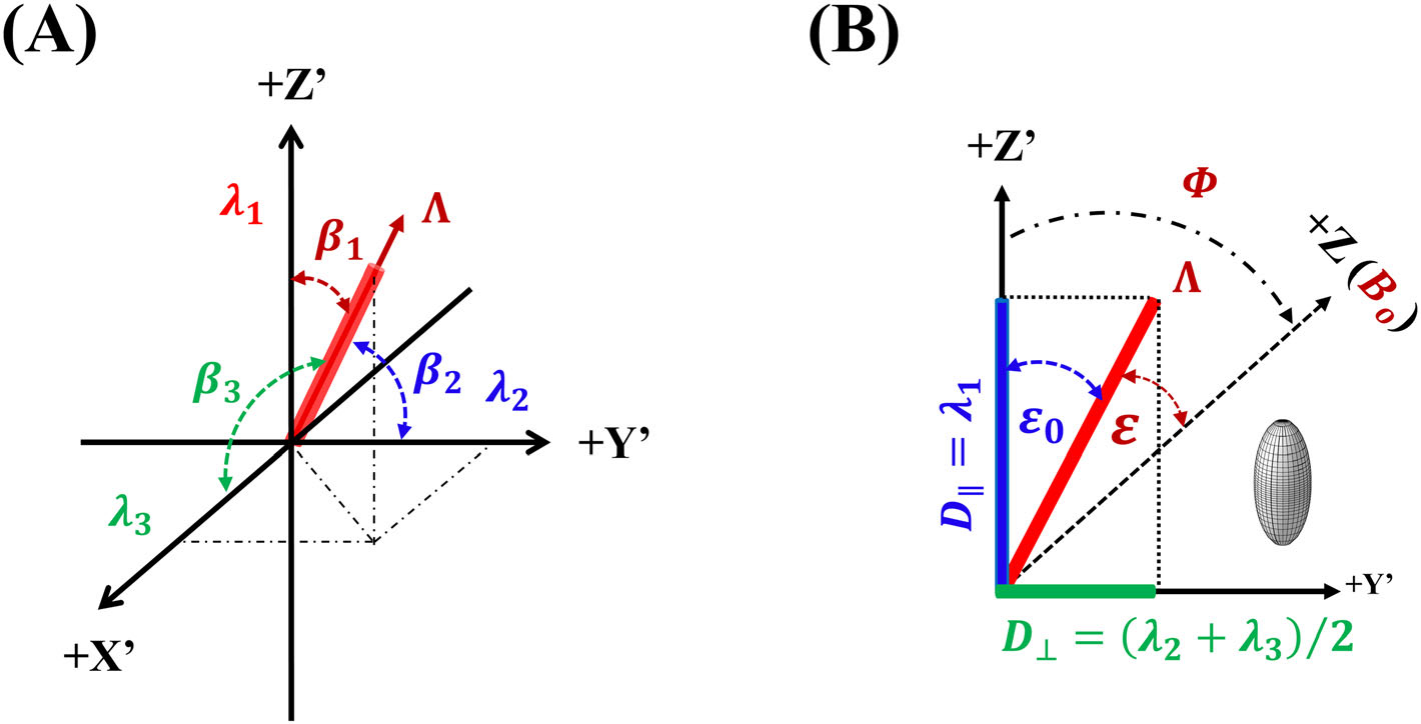
Definitions of an effective diffusion vector Λ in the principal axis system (A) and a phase shift $\varepsilon_{0}$ for an axially symmetric linear tensor (B). $\lambda_{1}$, $\lambda_{2}$, and $\lambda_{3}$ are, respectively, the primary or principal, secondary, and tertiary eigenvalues of the diagonalized diffusion tensor Λ.

An axon fiber orientation ε may be determined voxel‐wise by three eigenvalues $\lambda_{i}$ and three eigenvectors $e_{i}$ ( i=1,2,3), rather than by the principal diffusivity $\lambda_{1}$ direction $e_{1}$ as usual. From the laboratory reference frame (LRF) to the principal axis system (PAS), an apparent diffusion tensor ***D*** could be mathematically transformed into a diagonalized matrix Λ,^42^ which may be considered as a vector, as illustrated in Figure 2A. This vector has a magnitude $\Lambda =\sqrt{\lambda_{1}^{2}+\lambda_{2}^{2}+\lambda_{3}^{2}\;}$ and a direction signified by a unit vector, $e={\left(e_{x},e_{y},e_{z}\right)}^{T}$, as expressed in Equation (4).

(4) $$e=\cos\beta_{1}e_{1}+\cos\beta_{2}e_{2}+\cos\beta_{3}e_{3}$$

Here, $\cos\beta_{i}=\lambda_{i}/\Lambda$ is the direction cosine of the vector Λ; $e_{i}={\left(e_{\mathrm{ix}},e_{\mathrm{iy}},e_{\mathrm{iz}}\right)}^{T}$ is the direction cosine of each PAS orthonormal axis in the LRF (*i* = 1, 2, 3);^42^ bold face letters symbolize vectors or matrices throughout the text; and the letter *T* denotes the transpose operator.

Because D is composed of both anisotropic $D_{\text{aniso}}$ and isotropic $D_{\mathrm{iso}}$ components, with the latter being devoid of any orientation attributes, the direction of $D_{\text{aniso}}$ ought to be collinear with the unit vector e direction. This seemingly unusual interpretation of D is actually in accordance with the conclusion that D and $D_{\text{aniso}}$ share the same sets of eigenvectors, albeit with different eigenvalues.^42^ Accordingly, an actual axon fiber orientation ε must be determined by the anisotropic diffusivity direction (ADD) (i.e., $\varepsilon =\cos^{-1}e_{z}$). As a comparison, this fiber orientation has always been associated with the PDD in the literature,^8^, ^9^ that is, $\Phi =\cos^{-1}e_{1z}$, implying $\lambda_{1}\gg \lambda_{2}>\lambda_{3}$ or $\beta_{1}\approx 0{}^{\circ}$, an unlikely scenario for most of the imaging voxels from DTI. So, it comes as no surprise that previously reported $R_{2}^{*}$ and $R_{2}$ orientation dependences manifested a phase shift $\varepsilon_{0}$ as those observed in ex vivo studies, due to the difference between the actual ε and the measured Φ.

If directional diffusivities are available voxel‐wise or averaged across whole brain WM, $\varepsilon_{0}$ could be estimated for an axially symmetric linear tensor, as shown in Figure 2B. In this case, the two direction angles in the PAS become complementary (i.e., $\beta_{1}+\beta_{3}=90{}^{\circ}$). It is also valid for Φ (i.e., $\cos^{-1}e_{1z}$) in the LRF and its counterpart (i.e., $\cos^{-1}e_{3z}$). As a result, Equation (4) can be approximated by Equation (5a). Based on the cosine addition formula, ε will be estimated using Equation (5b), in which $\beta_{1}$ has been replaced by $\varepsilon_{0}=$ $\tan^{-1}\left(D_{⊥}/D_{∥}\right)$, with $D_{∥}=\lambda_{1}$ and $D_{⊥}=\left(\lambda_{2}+\lambda_{3}\right)/2$.

(5a) $$e_{z}\approx \cos\beta_{1}e_{1z}+\cos\beta_{3}e_{3z}$$

(5b) $$\varepsilon \approx \Phi -\varepsilon_{0}$$

### 2.4 $R_{2}$ orientation dependence from a single $T_{2}$‐weighted image

If the orientation information is available, anisotropic $R_{2}$ relaxation can be effectively quantified using only a single $T_{2}$‐weighted (T2W) image.^33^, ^43^ An imaging voxel signal SO with *b* = 0 in DTI may be expressed by Equation (6a), with $S_{0}$, TE, and $R_{2}^{\exp}$, respectively, denoting signal intensity with TE=0, spin‐echo time, and apparent transverse relaxation rate that could be decomposed into isotropic $R_{2}^{i}$ and anisotropic $R_{2}^{a}f\left(\alpha ,\varepsilon \right)$ components, as defined in Equation (1).^29^

(6a) $$\mathrm{SO}=S_{0}*\exp\left(-\mathrm{TE}*R_{2}^{\exp}\right)$$

(6b) $$\frac{\ln\mathrm{SO}}{\mathrm{TE}}=\left(\frac{\ln S_{0}}{\mathrm{TE}}-R_{2}^{i}\right)-R_{2}^{a}*f\left(\alpha ,\varepsilon \right)$$

On a logarithmic scale and further normalized by *TE*, Equation (6a) could be replaced by Equation (6b). Here, the parenthesized composite term (i.e., $\ln S_{0}/\mathrm{TE}-R_{2}^{i}$) may be considered as an unknown constant $C_{0}$ with a fixed *TE* in DTI. This is because both $S_{0}$ and $R_{2}^{i}$ reportedly hardly altered when compared with the variation of $R_{2}^{a}f\left(\alpha ,\varepsilon \right)$ across brain WM.^5^ It should be emphasized that $R_{2}^{i}$ cannot be determined with a single T2W image.

## 3 METHODS

### 3.1 Public domain $R_{2}^{*}$ and $R_{2}$ orientation dependences of brain WM

Unless stated otherwise, all $R_{2}^{*}$ and $R_{2}$ profiles presented herein were extracted from graph images of previous publications using a free online tool (www.graphreader.com). These extracted data were originally measured from the human brain WM in vivo or ex vivo at different $B_{0}$ fields, following ethical guidelines as stated in the original papers.

#### 3.1.1 $R_{2}^{*}$ orientation dependences of brain WM ex vivo at 7 and 11.7 T

**FIGURE 3:**
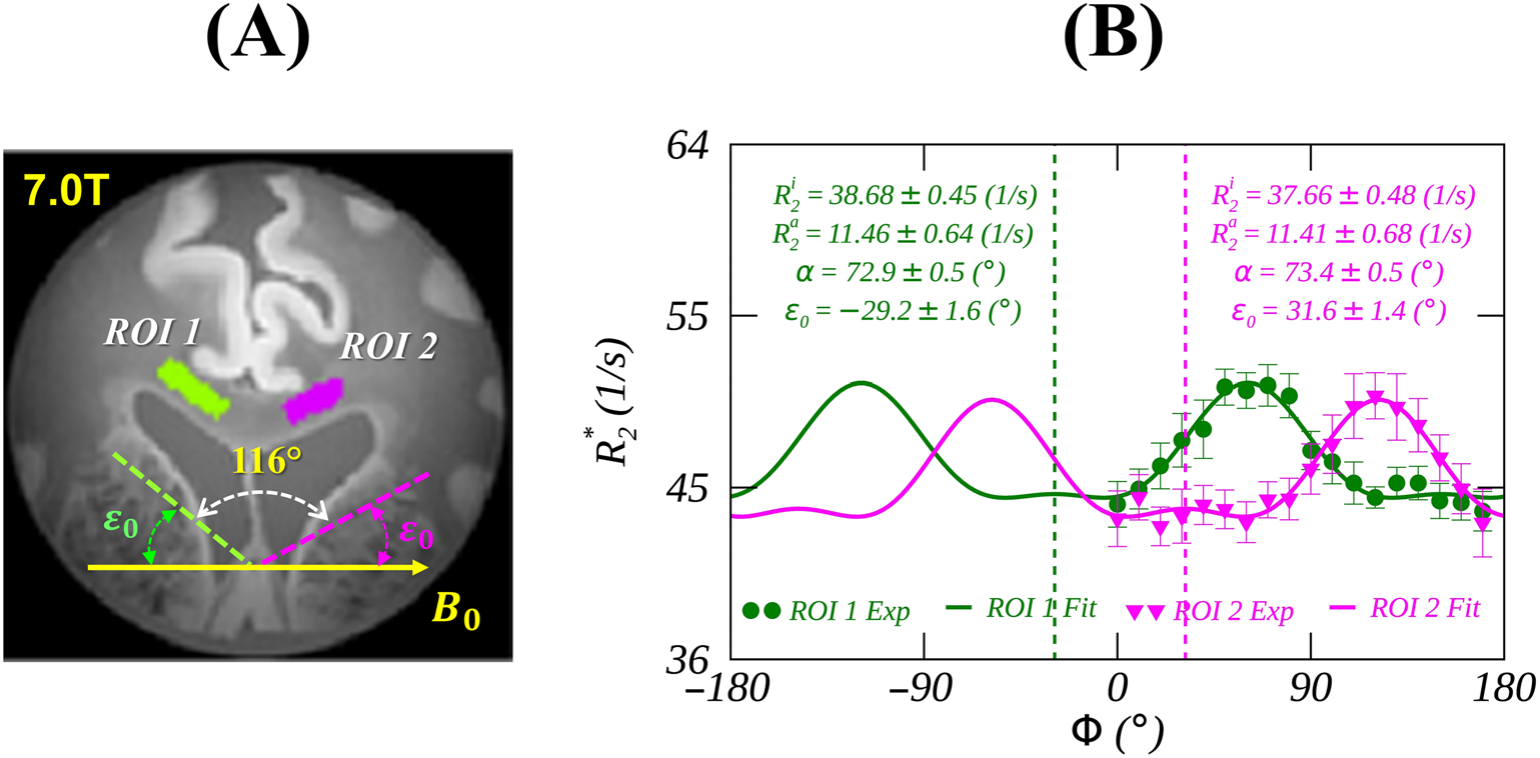
Two phase‐shifted $R_{2}^{*}$ orientation dependence profiles from a brain specimen at 7 T. (A) The brain image was adapted from fig. 2a and (B) The $R_{2}^{*}$ datasets were extracted from fig. 2b in the original publication.^12^

Two $R_{2}^{*}$ datasets were extracted from the human brain WM specimens.^11^, ^12^ The first was from a brain specimen (Sample 1) at 7 T, as presented in figs 2a‐b of the original paper,^12^ which were replotted (circle vs. triangle symbols) in Figure 3B in this work. These profiles were measured from two rectangular regions of interest (ROIs; green vs. magenta), as depicted in Figure 3A, including the major fiber bundles of the corpus callosum. This specimen was successively rotated from 0 ° to 170 ° relative to $B_{0}$ within a whole‐body MR scanner for 18 $R_{2}^{*}$ measurements.

**FIGURE 4:**
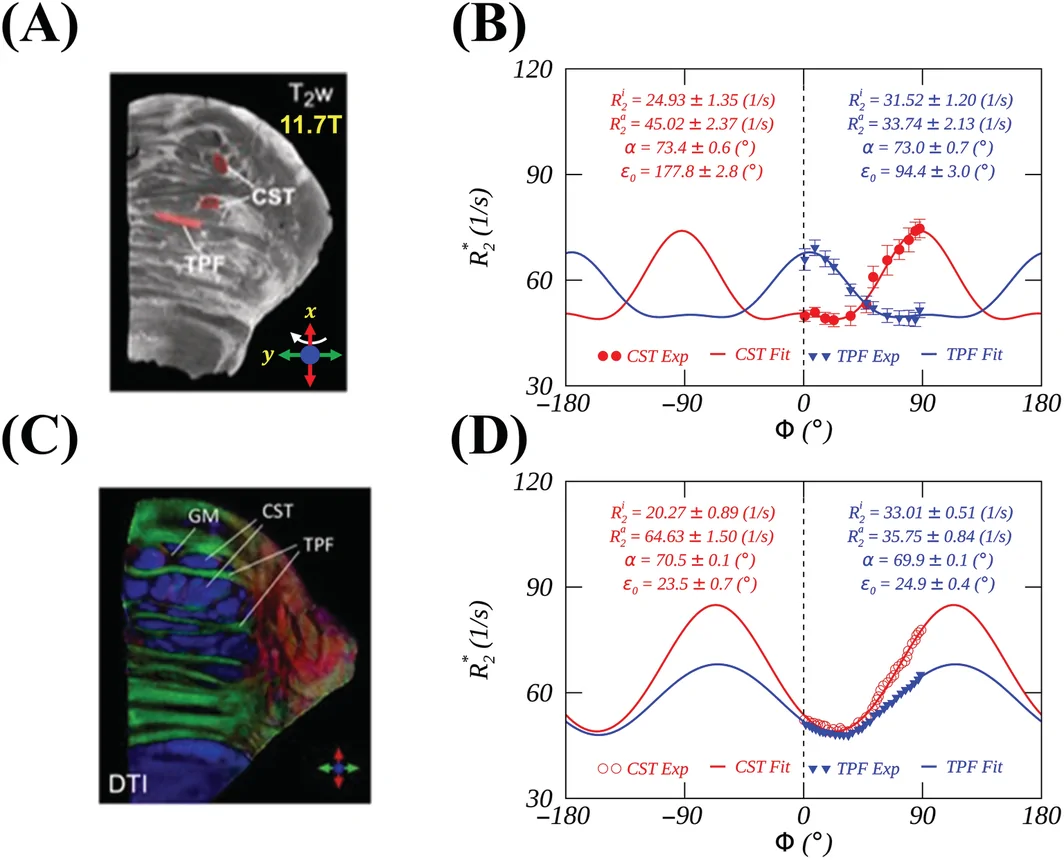
Two phase‐shifted $R_{2}^{*}$ orientation dependence profiles from a brainstem specimen at 11.7 T. Axon fiber orientations were either (A and B) Independent of or (C and D) Dependent on diffusion tensor imaging. The (A and C) brainstem images and (B and D) $R_{2}^{*}$ datasets were, respectively, adapted from figs 1 and 3 and extracted from fig. 3 in the original publication.^11^ CST, corticospinal tract; DTI, diffusion tensor imaging; GM, gray matter; TPF, transverse pontine fibers.

The second was derived from a fixed human brainstem specimen at 11.7 T, as shown in fig. 3 of the original publication.^11^ The extracted $R_{2}^{*}$ profiles of the corticospinal tract (CST; red circles) and transverse pontine fibers (TPF; blue triangles) were replotted in Figure 4B,D in this work. While the CST run along the foot–head direction in the brain, the TPF were approximately aligned mediolaterally and thus perpendicular to the CST. The fiber orientations in Figure 4B were manually measured based on the long axis of ROIs relative to the $B_{0}$ direction, as illustrated in Figure 4A. By contrast, the fiber orientations in Figure 4D were indirectly determined based on the standard PDD from DTI, as seen in Figure 4C. As the measured $R_{2}^{*}$ values associated with DTI‐based orientations were presented in scatterplots in the original paper, it was challenging to replicate original data with high confidence. Nonetheless, the extracted $R_{2}^{*}$ datapoint for each orientation in Figure 4D was a best estimate for an average from several $R_{2}^{*}$ values at the angle.

#### 3.1.2 $R_{2}^{*}$ and $R_{2}$ orientation dependences of brain WM in vivo at 3 and 7 T

**FIGURE 5:**
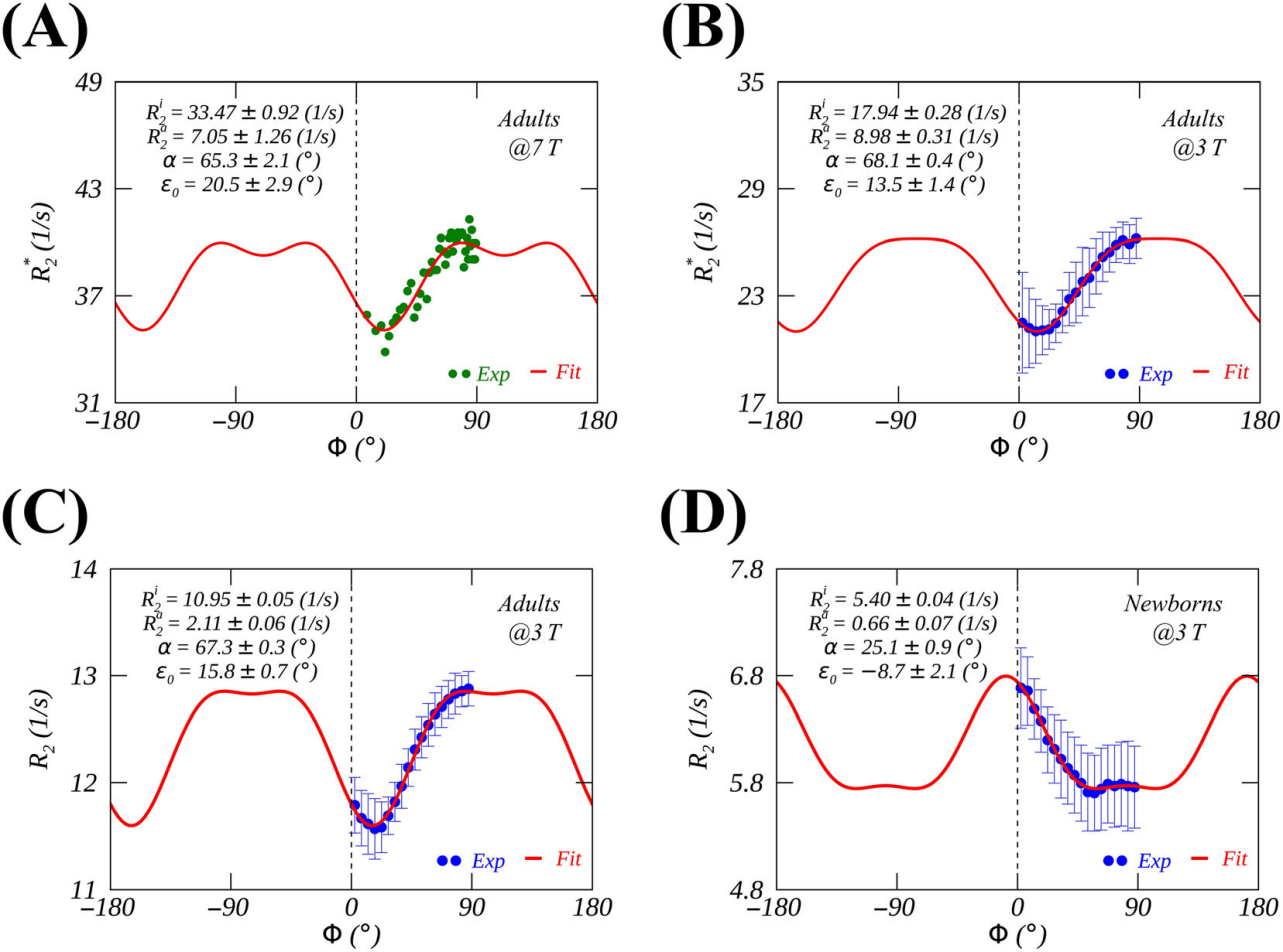
Phase‐shifted $R_{2}^{*}$ (A and B) and $R_{2}$ (C and D) orientation dependence profiles in the brain white matter from either (A‐C) Adults or (D) Newborns at either (A) 7 T or (B‐D) 3 T. These $R_{2}^{*}$ and $R_{2}$ datasets were extracted from three original publications.^2^, ^17^, ^44^

Four transverse relaxation orientation dependence datasets were extracted from previously published in vivo studies^2^, ^17^, ^44^ and were replotted consecutively in Figure 5A‐D in this work. The first was $R_{2}^{*}$ profile at 7 T without the confounding flip angle effect.^44^ This dataset was measured from three healthy subjects (age 38.7 ± 4.2 years), as shown in fig. 7 (Session2, green) from the original paper. The second was the $R_{2}^{*}$ profile at 3 T from four healthy subjects (age 29.5 ± 5.6 years), reproduced from fig. 2a (red triangles) in the original work.^2^ The third and the fourth were $R_{2}$ orientation dependences at 3 T from eight healthy adults (age 21 ± 2 years) and eight newborns (age 40.4 ± 1.1 weeks), replicated from fig. 6 in the supplementary information of the original paper.^17^

#### 3.1.3 $R_{2}$ orientation dependences from a single T2W image

The origin of the phase shift $\varepsilon_{0}$ associated with DTI was revealed on ultrahigh resolution Connectome DTI datasets from one healthy subject (age = 30 years).^45^ With an isotropic voxel size of 760 μm ^3^, the reconstructed image matrix was 292 (left–right) by 288 (anterior–posterior) by 192 (superior–inferior). Paired with *b* = 0 (s/mm^2^) images, six preprocessed data subsets with *b* = 1000 (s/mm^2^) and 12 preprocessed data subsets with *b* = 2500 (s/mm^2^) were individually analyzed using FSL DTIFIT.^46^ The outputs were as follows: (a) $T_{2}$‐weighted signal SO with *b* = 0, from which an anisotropic $R_{2}$ profile was derived based on Equation (6b); (b) eigenvalues $\lambda_{i}$ and eigenvectors $e_{i}$ ( i= 1, 2, 3); (c) fractional anisotropy (FA); and (d) mode of anisotropy (MO),^47^ with MO=1 denoting an ideal linear diffusion tensor for one fiber population within an imaging voxel, and with MO=−1 indicative of a perfect planar diffusion tensor for crossing or fanning fiber tracts. In this work, WM voxels were categorized by 0.5<FA<0.9 and 0.5<MO<1.0.

### 3.2 A unified $R_{2}^{*}$ and $R_{2}$ model with a phase shift $\varepsilon_{0}$

Equation (1) and Equation (6b) may be consolidated into Equation (7), which comprises four model parameters, that is, constant $C_{0}$, $R_{2}^{a}$, α, and a phase shift $\varepsilon_{0}$.

(7) $$y=C_{0}\pm R_{2}^{a}*f\left(\alpha ,x-\varepsilon_{0}\right)$$

The plus sign was applied when the dependent variable y denoted relaxation rates, as written in Equation (1); otherwise, the minus sign was used when y was associated with T2W signals, as expressed in Equation (6b). In the first scenario, $C_{0}$ simply became $R_{2}^{i}$, and the independent variable x was represented by Φ, denoting orientations either measured manually or determined indirectly by the PDD from DTI. In the second case, $C_{0}$ was equivalent to $\ln S_{0}/\mathrm{TE}-R_{2}^{i}$ with *TE* = 75 ms,^45^ and x was represented either by ε from the ADD as theorized in this work or by Φ from the PDD as usual.

**FIGURE 6:**
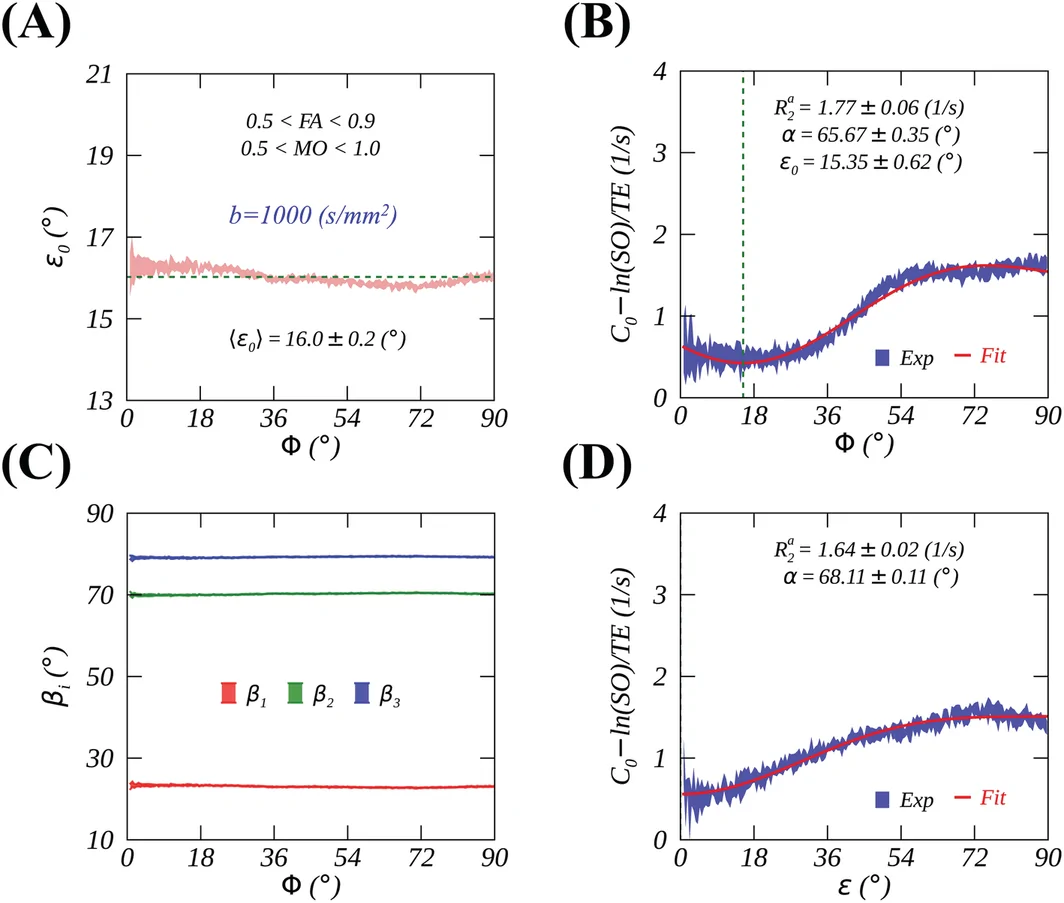
An anisotropic $R_{2}$ profile from T2W (*b* = 0) based on directional diffusivities from *b* = 1000 (s/mm^2^) DTI data subsets (B) With and (D) Without a phase shift $\varepsilon_{0}$. Also presented are (A) Voxel‐wise orientation‐dependent $\varepsilon_{0}$ and (C) Direction angle $\beta_{i}$ ( i = 1, 2, 3). These results were based on public domain DTI datasets.^45^ DTI, diffusion tensor imaging; T2W, $T_{2}$‐weighted.

**FIGURE 7:**
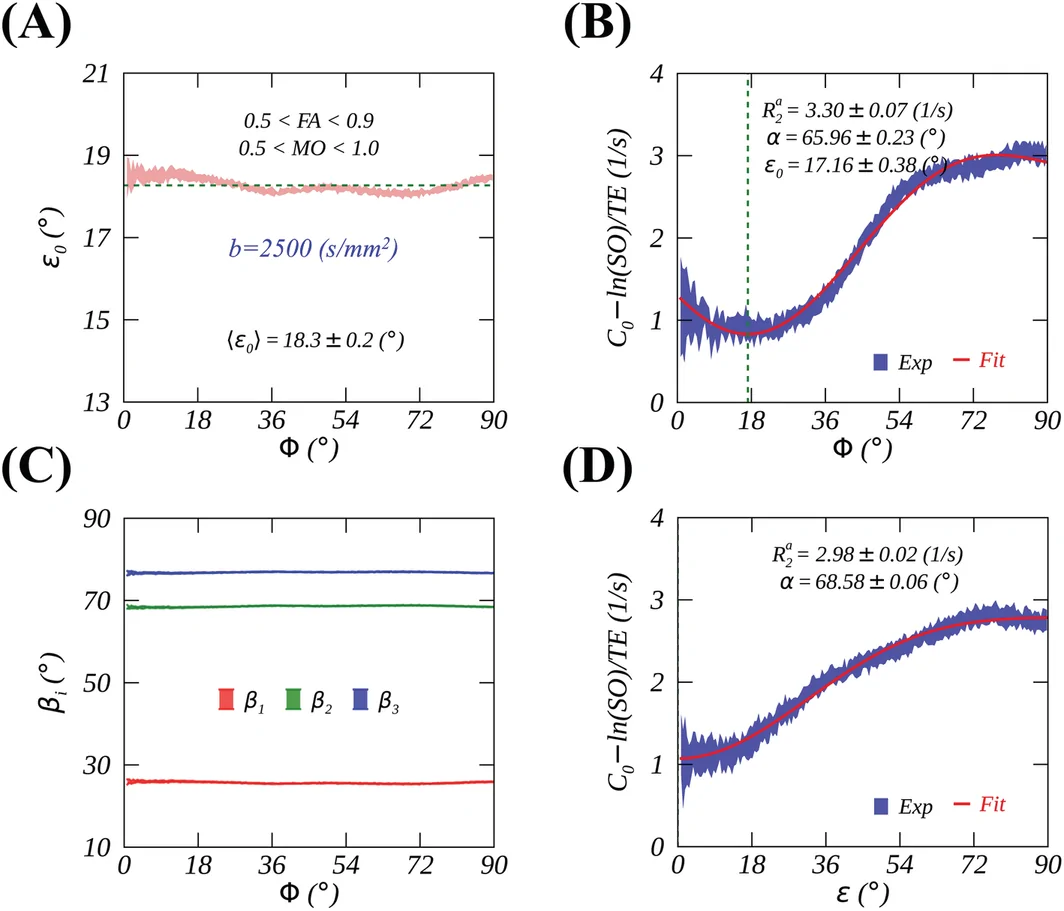
An anisotropic $R_{2}$ profile from T2W (*b* = 0) based on directional diffusivities from *b* = 2500 (s/mm^2^) DTI data subsets (B) With or (D) Without a phase shift $\varepsilon_{0}$. Also presented are (A) Voxel‐wise orientation‐dependent $\varepsilon_{0}$ and (C) Direction angle $\beta_{i}$ ( i= 1, 2, 3). These results were based on public domain DTI datasets.^45^ DTI, diffusion tensor imaging; T2W, $T_{2}$‐weighted.

The calculated ε and Φ for brain WM voxels were sorted ascendingly from 0° to 90° following the literature,^5^, ^8^, ^9^ and then averaged within each predefined data bin (size = 0.5°) to generate an orientation dependence profile for associated T2W signals as well as some diffusion metrics. Except for those sorted based on ε in Figure 6D and Figure 7D, the orientation‐dependent variables that appeared in all the figures were dependent on Φ just for consistency with the literature.

### 3.3 Nonlinear least‐squares curve fittings

A robust Levenberg–Marquardt nonlinear least‐squares optimization method, from a public domain (http://purl.com/net/mpfit),^48^ was used for data modeling with constrained model parameters. The fittings were either weighted or unweighted depending on availabilities of measurement uncertainties denoted by error bars in all plots. For the unweighted case, a unity error was applied to all measured data. The uncertainties of fitted model parameters were rescaled so that the reduced $\chi^{2}$ became unity.

$f\left(\alpha ,x-\varepsilon_{0}\right)$ was parameterized in the factored form unless otherwise specified, and the fitted model parameters were $R_{2}^{i}$ or $C_{0}$, $R_{2}^{a}$, α, and $\varepsilon_{0}$. When $f\left(\alpha ,x-\varepsilon_{0}\right)$ was in the expanded form, the fitted parameters became $C_{0}$, $C_{1}$, $C_{2}$, and $\varepsilon_{0}$. Generally, these model parameters were constrained to the following ranges: $R_{2}^{i}$= $C_{0}$ = [0, 100] (1/s), $R_{2}^{a}$ = $C_{1}$= [0, 50] (1/s), and α = [0°, 90°] or $C_{2}$ = [0, 50] (1/s). If the constraints were not set to non‐negative numbers, $C_{1}$ and $C_{2}$ were optimized within the range [−50, 50] (1/s). As for the phase shift $\varepsilon_{0}$, its constraint was typically set to [0°, 90°] but could be altered for some cases. For instance, the range of $\varepsilon_{0}$ was extended to [−180°, 180°] to cover a larger phase shift for one of two orthogonal axon fiber tracts in Figure 4B.

Divided into five subgroups, the maximum number of iterations was set to 1000. To avoid being trapped within local minima during optimization processes, each subgroup started with unique initial values within the constraints. In this work, the fitted y, labeled as “ Fit”, was plotted using colored solid lines, and the fitted model parameters were imprinted on the graphs for easy reference. Further, the standard deviations of measured (i.e., “ Exp”) data were denoted by error bars or half widths of shaded ribbons. A goodness of fit was quantified by the root‐mean‐square error normalized by the mean value of measurements ( NRMSE%). An F‐test was used for comparing two fittings with and without $\varepsilon_{0}$. Statistical significance was considered to be a *p* of 0.05 or less. All data analysis and visualization were conducted using in‐house software developed in IDL 8.8 (Harris Geospatial Solutions, Inc., Broomfield, CO, USA).

## 4 RESULTS

### 4.1 Theoretical $f\left(\alpha ,\varepsilon \right)$ profiles in brain WM

Figure 1B shows five $f\left(\alpha ,\varepsilon \right)$ profiles for fiber orientations −180°≤ε≤180° when angles α= 0 ° (red), 20 ° (green), 45 ° (blue), 70 ° (black), and 90 ° (magenta). Figure 1D focuses on these profiles ( 0°≤ε≤90°), showing different $∆f\left(\alpha ,\varepsilon \right)$ as α varies, for example, $∆f\left(0{}^{\circ},\varepsilon \right)=4$ and $∆f\left(90{}^{\circ},\varepsilon \right)\approx 1$. Here, $∆f\left(\alpha ,\varepsilon \right)$ was calculated as the difference between the maximum and the minimum of $f\left(\alpha ,\varepsilon \right)$ values. A full picture of $f\left(\alpha ,\varepsilon \right)$ is presented in Figure 1C, revealing multiple symmetries embedded in an axially symmetric model.

### 4.2 $R_{2}^{*}$ orientation dependences of brain WM ex vivo

Figure 3B shows the shifted $R_{2}^{*}$ profiles at 7 T from two rectangular ROIs in the corpus callosum, as depicted in Figure 3A. Although the fitted $R_{2}^{i}$, $R_{2}^{a}$, and α values were all consistent, the fitted $\varepsilon_{0}$ turned out to be completely different, that is, with $\varepsilon_{0}=-29.2\pm 1.6{}^{\circ}$ (vertical green line, ROI1) for the left shifted profile and with $\varepsilon_{0}=31.6\pm 1.4{}^{\circ}$ (vertical magenta line, ROI2) for the right shifted profile. These fitting results indicate that the initial orientations of ROI long axes were not aligned along $B_{0}$, as illustrated in Figure 3A. The angle between these two long axes was $180{}^{\circ}-\left(31.6{}^{\circ}+29.2{}^{\circ}\right)=119.2{}^{\circ}$, approximately equal to 116 ° measured using a protractor on the graph.

For the brainstem specimen at 11.7 T, the longitudinal fascicles of the CST and TPF were orthogonally interweaved, as seen in Figure 4A,C. When the orientation information was derived by manually rotating the specimen, the $R_{2}^{*}$ profiles from the two axon fiber tracts were out of phase by roughly 90 °, as revealed in Figure 4B, that is, $\varepsilon_{0}=177.8\pm 2.8{}^{\circ}$ (CST, red) versus 94.4±3.0° (TPF, blue). However, when the orientation information was determined by the PDD from DTI, the two $R_{2}^{*}$ profiles became in phase but right shifted by $\varepsilon_{0}\approx 24{}^{\circ}$, as revealed in Figure 4D.

### 4.3 $R_{2}^{*}$ and $R_{2}$ orientation dependences of brain WM in vivo

As shown in Figure 5A,B, $R_{2}^{*}$ profiles of brain WM from adults manifested considerable right phase shifts at both 7 T (i.e., $\varepsilon_{0}=20.5\pm 2.9{}^{\circ}$) and 3 T (i.e., $\varepsilon_{0}=13.5\pm 1.4{}^{\circ}$). Similarly, the $R_{2}$ profile from adults was also right shifted at 3 T (i.e., $\varepsilon_{0}=15.8\pm 0.7{}^{\circ}$), as seen in Figure 5C. Within measurement errors, the fitted $R_{2}^{a}$ (1/s) from $R_{2}^{*}$ profiles were comparable at two $B_{0}$ fields (i.e., 7.05 ± 1.26 vs. 8.98 ± 0.31). However, these $R_{2}^{a}$ values were much larger than that (i.e., 2.11 ± 0.06) from the $R_{2}$ profile at 3 T. Nonetheless, the fitted α were all consistent in these cases. As revealed in Figure 5D, the $R_{2}$ profile from newborns at 3 T was completely different when compared with those from adults, signified by unusual model parameters, that is, $\varepsilon_{0}=-8.7\pm 2.1{}^{\circ}$, $R_{2}^{a}=0.66\pm 0.07$ (1/s), and α=25.1±0.9°.

**FIGURE 8:**
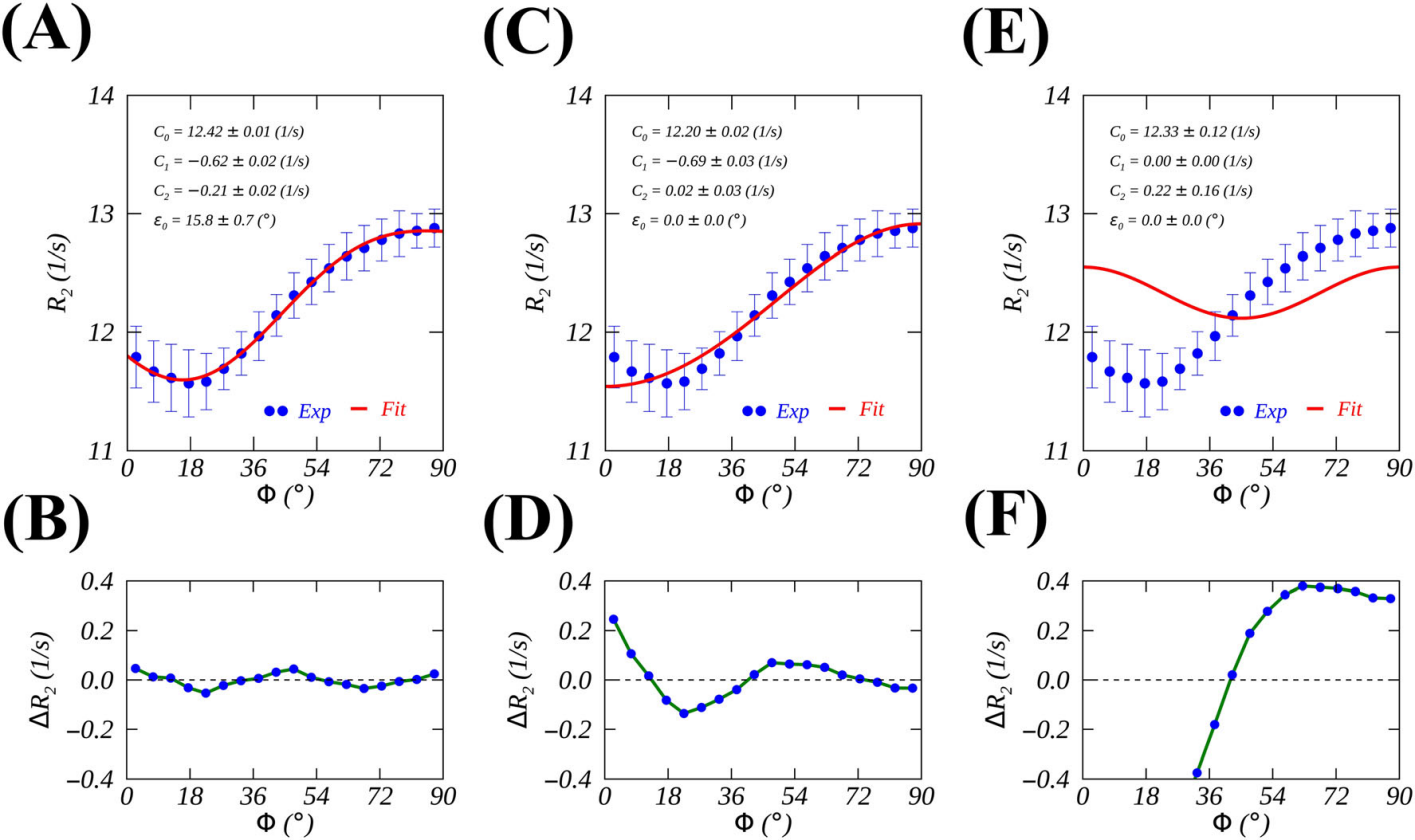
Phase‐shifted $\;R_{2}$ profile from Figure 5C quantified by different model parameters: (A and C) Without or (E) With non‐negative number constraints, and (C and E) Without or (A) With a phase shift $\varepsilon_{0}$. The associated fitting residues (i.e., $∆R_{2}=\mathrm{Exp}-\mathrm{Fit}$) are shown consecutively in (B), (D), and (F). For the purposes of verification, from Figure 5C, { $R_{2}^{i}$ (1/s), $R_{2}^{a}\left(1/s\right)$, $\alpha \left({}^{\circ}\right)$} were equal to {10.95, 2.11, 67.3} and then α‐dependent { $A_{0}$, $A_{1}$, $A_{2}$} became {0.696, −0.293, −0.097}. By definitions, $C_{0}=R_{2}^{i}+R_{2}^{a}*A_{0}=10.95+2.11*0.696=12.42\;\left(1/s\right)$, $C_{1}=R_{2}^{a}*A_{1}=2.11*\left(-0.293\right)=-0.62\;\left(1/s\right)$, and $C_{2}=R_{2}^{a}*A_{2}=2.11*\left(-0.097\right)=-0.21\left(1/s\right)$, as shown in (A).

With respect to those fits without $\varepsilon_{0}$ (i.e., $\varepsilon_{0}=0{}^{\circ}$) as usually found in the literature, the fitting residuals for the presented (i.e., $\varepsilon_{0}\neq 0{}^{\circ}$) profiles were all reduced significantly ( p<0.01), except for that in Figure 5A (i.e., NRMSE%=1.75 vs1.92, p=0.56). For instance, Figure 8A highlights an enlarged portion of Figure 5C within the measured orientation range, and the fitting residuals (i.e., $∆R_{2}\;$= Exp – Fit) are shown in Figure 8B. For this $R_{2}$ dataset, the fitted parameters were $C_{0}$, $C_{1}$, and $C_{2}$, as imprinted in Figure 8A, that corresponded to $R_{2}^{i}$, $R_{2}^{a}$, and α, as seen in Figure 5C. From Figure 8B with $\varepsilon_{0}=15.8\pm 0.7{}^{\circ}$ to Figure 8D with $\varepsilon_{0}=0{}^{\circ}$ (fixed), the fitting residuals increased significantly in terms of NRMSE% (i.e., 0.22 vs. 0.71, p<0.01). When $C_{1}$ had been further constrained to a non‐negative number, the corresponding fit deviated more from the measurement, as demonstrated in Figure 8E,F.

### 4.4 Anisotropic $R_{2}$ of brain WM from a single T2W image

Figure 6 presents analysis results from six DTI data subsets with *b* = 1000 (s/mm^2^). More specifically, an orientation‐dependent phase shift $\varepsilon_{0}$ is plotted in Figure 6A, derived from voxel‐wise directional diffusivities by $\tan^{-1}\left(D_{⊥}/D_{∥}\right)$. An average phase shift $\left\langle \varepsilon_{0}\right\rangle$ across all brain WM voxels was around 16.0°, as indicated by a horizontal dashed green line. As demonstrated in Figure 6B, this predicted $\left\langle \varepsilon_{0}\right\rangle$ value was equal to the fitted $\varepsilon_{0}=15.4\pm 0.6{}^{\circ}$ (vertical dashed green line) within measurement errors. Similar results from a previous study^4^ also confirmed the relationship between the fitted $\varepsilon_{0}$ and directional diffusivities from DTI (data not shown).

If an axon fiber angle ε was determined by three diffusivities $\lambda_{i}$ (not shown) and three direction angles $\beta_{i}$ ( i=1,2,3), as seen in Figure 6C, the revised $R_{2}$ profile as seen in Figure 6D became free from a confounding phase shift, but with comparable fitted $R_{2}^{a}$ and α values. Although the $R_{2}$ profile in Figure 6B was derived from T2W signals, its fitted model parameters were still consistent with those from Figure 5C, rendering strong support to the assumption used in this work, that is, unchanged $S_{0}$ and $R_{2}^{i}$ across WM in the human brain.

Figure 7 demonstrates the comparable results from 12 DTI data subsets with *b* = 2500 (s/mm^2^). In particular, the predicted and the fitted $\varepsilon_{0}$ were still consistent, as revealed in Figure 7A,B (i.e., 18.3±0.2° vs. 17.2±0.4°), albeit slightly larger than those observed in Figure 6. When an axon fiber orientation was determined by the proposed ADD, the $R_{2}$ profile was not confounded by $\varepsilon_{0}$, as indicated in Figure 7D. By contrast, this profile was right shifted when the axon fiber orientation relied on the standard PDD, as shown in Figure 7B.

## 5 DISCUSSION

This work has demonstrated that a ubiquitous phase shift $\varepsilon_{0}$ is a prerequisite for better quantifying $R_{2}$ and $R_{2}^{*}$ orientation dependences in the human brain WM. A phase‐shifted generalized transverse relaxation model was used to characterize some previously published datasets from both ex vivo and in vivo studies at different *B_0_* fields. Furthermore, the origin of $\varepsilon_{0}$ associated with DTI has been elucidated and a quantitative relationship between $\varepsilon_{0}$ and the directional diffusivities has also been established.

### 5.1 An intrinsic phase shift $\varepsilon_{0}$ in $R_{2}$ and $R_{2}^{*}$ profiles

When an axon fiber orientation was determined by the proposed ADD rather than by the standard PDD, as demonstrated in Figures 6D and 7D, the phase shift $\varepsilon_{0}$ once manifested in the $R_{2}$ profile disappeared. This result clearly demonstrates the origin of $\varepsilon_{0}$ as predicted in the current work. From a different perspective, a phase‐shifted anisotropic transverse relaxation profile in brain WM has the hallmark of an intimately linked anisotropic translational diffusion characteristic, that is, a smaller $\varepsilon_{0}$ indicative of a larger FA according to $\tan\varepsilon_{0}=D_{⊥}/D_{∥}$.

It is obvious that ex vivo $R_{2}^{*}$ profiles in Figure 3B cannot be adequately characterized without $\varepsilon_{0}$; however, the essence of $\varepsilon_{0}$ has not yet been fully revealed in the literature.^12^ Initially, two phase offsets or shifts (i.e., $\varphi_{0}$ and $\varphi_{1}$) were applied to the model function combining both sin2θ and sin4θ terms. These two trigonometric functions were integrated by simultaneously considering magnetic susceptibility perturber structure and magnetic susceptibility anisotropy structure. The phase coherence between the two offsets was later uncovered, and then only one phase shift $\varphi_{0}$ was defined for both trigonometric functions such as $\sin\left(2\theta +\varphi_{0}\right)$. This phase shift definition is slightly different from ours for $\varepsilon_{0}$ in the same term $\sin\left(2\left(\theta -\varepsilon_{0}\right)\right)$. Interestingly, this essential phase parameter has not been explicitly considered in ex vivo data modeling,^10^ and was even completely ignored for an in vivo study in the following publication.^21^ It would be fair to state that the significance of $\varepsilon_{0}$ has not yet been fully appreciated to date, particularly in those studies where DTI is utilized for obtaining the information about axon fiber orientations.

Whether the orientation information was obtained directly by manually rotating brain WM specimens or derived indirectly from DTI, the fitted model parameters (except $\varepsilon_{0}$) ought to be consistent. To some degree, this was the case for the TPF (blue), as shown in Figure 4, but was not the case for the CST (red), particularly for the fitted $R_{2}^{a}$(1/s), that is, 45.0 versus 64.6. These observed discrepancies between the fits in Figure 4B and those in Figure 4D could be explained in part by imprecise data in Figure 4D, which were extracted from original scatterplots. Nonetheless, a phase shift is undoubtedly evident and becomes indispensable in better characterizing $R_{2}^{*}$ profiles, irrespective of the origin of the axon fiber orientation information.

### 5.2 Biophysically meaningful model parameters

The theoretical transverse relaxation orientation dependence function $f\left(\alpha ,\varepsilon \right)$ can be parameterized either by a factored form or by an expanded form. When comparing Equation (2) with Equation (3), the former appears to be more complex than the latter; in fact, the data modeling using either of the two has the same fitting performance in terms of fitting residuals, albeit with different model parameters (except $\varepsilon_{0}$). For instance, the previously measured $R_{2}^{*}\left(1/s\right)$ from ROI2 (magenta) in a brain WM specimen (Figure 3B) can be equally described by the model functions $37.7+11.4f\left(73.4{}^{\circ},\Phi -\varepsilon_{0}\right)$ and $45.9-3.0\cos2\left(\Phi -\varepsilon_{0}\right)+1.3\cos4\left(\Phi -\varepsilon_{0}\right)$. For these two model functions, the $\text{NRMSD}\left(\%\right)$ values were all equal to 0.99 and the fitted $\varepsilon_{0}$ was 31.6±1.4°.

While the fitted model parameters based on the factored form of $f\left(\alpha ,\varepsilon \right)$ were always positive, those based on the expanded form (see Figure 8) and its alternatives could become negative numbers. For example, the $R_{2}$ profile from Figure 8C could be alternatively modeled by $11.54+1.18\sin^{2}\Phi +0.19\sin^{4}\Phi$. However, if $\varepsilon_{0}$ was further incorporated for an optimal fit, as demonstrated in Figure 8A, this alternative function would become $11.6+2.87\sin^{2}\left(\Phi -15.8{}^{\circ}\right)-1.64\sin^{4}\left(\Phi -15.8{}^{\circ}\right)$, with one negative parameter. It is challenging to interpret these negative parameters in any biophysically meaningful context.

Because of the symmetries in $f\left(\alpha ,\varepsilon \right)$ and an inevitable $\varepsilon_{0}$, the $R_{2}$ or $R_{2}^{*}$ profile could be quantified equally well using different sets of model parameters, depending on the choice of the constraint of $\varepsilon_{0}$. For instance, if the limits of $\varepsilon_{0}$ were altered from the default [0 °, 90 °] to [90 °, 180 °], the $R_{2}\left(1/s\right)$ profile from Figure 8A could be characterized by $12.4+0.62\cos2\left(\Phi -\varepsilon_{0}\right)-0.21\cos4\left(\Phi -\varepsilon_{0}\right)$, with the fitted $\varepsilon_{0}=105.8\pm 0.7{}^{\circ}.$ In this scenario, $\varepsilon_{0}$ specified the orientation where the maximum $R_{2}$, rather than the minimum $R_{2}$ (as usual for adult brain WM), was observed. This dilemma of the model parameter degeneracy can, in general, be avoided if the default limits of $\varepsilon_{0}$ are kept for in vivo studies.

Nevertheless, both $R_{2}$ and $R_{2}^{*}$ profiles are constructed based on the averaged and sorted voxel‐wise data, and most of these data across the whole brain WM are aggregated into an orientation range between 30 ° and 90 ° relative to the $B_{0}$ direction. Consequently, neither the $R_{2}$ profile nor the $R_{2}^{*}$ profile can provide any localized microstructural information, even though the localized fiber tracts may have different physical characteristics such as varying axon fiber diameters. Thus, it is critical to keep this limitation in mind when interpreting the derived model parameters in any meaningful way.

### 5.3 Phase‐shifted $R_{2}^{*}$ profile from adults at 7 T and $R_{2}$ profile from newborns at 3 T

As revealed in Figure 5A, the fitting residuals with $\varepsilon_{0}$, in terms of NRMSE%, were insignificantly reduced compared with those without $\varepsilon_{0}$; nonetheless, this shifted model is compatible with a previously reported $R_{2}^{*}$ orientation dependence at 7 T. In the current work, the shifted $R_{2}^{*}$ profile manifested a minimum or a dip at Φ≈20°, while the same feature could be identified from fig. 2A in the literature,^49^ where an apparent $R_{2}^{*}$, averaged between the orientations 0–18 ° and between the orientations 18–36 °, were comparable. Similarly, the maximum $R_{2}^{*}$ value was found at angle Φ≈79° in Figure 5A from this work, which is consistent with $R_{2}^{*}$ changes from successive intervals of 36–54 °, 54–72 °, and 72–90 °, as deduced from fig. 2A in the literature.^49^

The $R_{2}$ profile from newborns at 3 T, as shown in Figure 5D, is completely different from that previously reported in the literature.^18^ Specifically, the measured $R_{2}$ shown herein and $T_{2}$ profiled in fig. 5 from the literature shared a similar orientation dependence trend, apparently contradictory from each other. Furthermore, the $R_{2}$ profile in Figure 5D considerably deviated from a previously reported $R_{2}^{*}$ profile from the same cohort of newborns at 3 T.^4^ Considering the similarity between $R_{2}^{*}$ and $R_{2}$ profiles from healthy adults, as demonstrated in Figure 5B,C, it becomes more challenging to uncover the origin of $R_{2}$ orientation dependence in newborns. Nevertheless, the standard MAE has been reportedly responsible for this unusual $R_{2}$ profile,^17^ which is a special case of the proposed orientation dependence function $f\left(\alpha ,\varepsilon \right)$ with α=0° and without a phase shift $\varepsilon_{0}$.

### 5.4 Origins of $R_{2}$ and $R_{2}^{*}$ orientation dependences in brain WM

When comparing Figure 7 with Figure 6, the fitted $R_{2}^{a}$ appeared relatively larger with a higher *b*‐value. In this work, the FA threshold for classifying brain WM voxels was kept the same regardless of varying *b*‐values. Because of an inverse correlation between *b*‐value and FA,^50^ more imaging voxels with a lower FA would be disqualified for WM voxels when using a higher *b*‐value. This would lead to a relatively larger average FA for the selected voxels compared with those with a lower *b*‐value. In other words, with a fixed FA threshold, the selected WM voxels appear more anisotropic (i.e., an increased $R_{2}^{a}$) for higher *b*‐values.

Because no consensus on either FA threshold or *b*‐value was available for previously published $R_{2}$ and $R_{2}^{*}$ orientation dependences in brain WM, it is no surprise that some inconsistent results can be found in the literature. For example, the $R_{2}^{a}$ (1/s) value from Figure 5C derived from spin‐echo signals at 3 T was 2.11 ± 0.06 associated with *b* = 700 (s/mm^2^), and it became 1.77 ± 0.06 (Figure 6B) associated with *b* = 1000 (s/mm^2^). Similarly, the $R_{2}^{a}$ (1/s) values from anisotropic $R_{2}^{*}$ profiles markedly differed (data not shown) even using the same *b* = 1000 (s/mm^2^) at 3 T,^5^, ^8^ which were much lower than those observed with *b* = 2000 (s/mm^2^), as shown in Figure 5B. These observations are consistent with the increased $R_{2}^{a}$ values from *b* = 1000 to 2500 (s/mm^2^), as demonstrated in Figures 6B and 7B, which were based on anisotropic $R_{2}$ orientation dependences. It should be mentioned that the DTIFIT outputs at *b*‐values approximately higher than 1000 (s/mm^2^) could be somewhat compromised because of the potential contribution of kurtosis.^51^ Thus, care should be exercised when interpreting these $R_{2}^{a}$ values at higher *b*‐values.

Besides its dependence on *b* values and FA thresholds, $R_{2}^{a}$ could also be impacted by imperfect slice profiles in 2D image acquisitions,^52^, ^53^ or by varying refocusing pulse intervals,^54^ or by temperature differences,^10^ as well as different flip angles.^44^ Without eliminating these confounding factors from $R_{2}^{a}$, it is unlikely to unravel the underlying relaxation mechanisms in $R_{2}^{*}$ and $R_{2}$ orientation dependences in brain WM. In the current work, different $R_{2}^{a}$ values were found from $R_{2}^{*}$ (Figure 5B) and $R_{2}$ (Figure 5C) profiles at 3 T, seemingly suggesting that the orientation dependence comes at least in part from $R_{2}^{\prime }$. However, the fact of the comparable $R_{2}^{a}$ values from $R_{2}^{*}$ profiles at 7 T (Figure 5A) and 3 T (Figure 5B) becomes contradictory to this tentative conclusion. In the literature,^31^ the decay of transverse magnetization in highly ordered tissues ex vivo was shown to be biexponential, with components varying at different angles and at different $B_{0}$ fields; therefore, the monoexponential approximation could be the culprit for the observed contradiction. Nonetheless, the proposed theoretical framework can still be valuable even without knowing the exact relaxation mechanisms involved, because the proposed generalized transverse relaxation model is directly linked to the axon fiber microstructures in brain WM.

## 6 CONCLUSIONS

A phase shift $\varepsilon_{0}$ in a proposed model function has been revealed as essential for better quantifying $R_{2}$ and $R_{2}^{*}$ orientation dependences in brain WM. Furthermore, the origin of $\varepsilon_{0}$ associated with directional diffusivities from DTI has been elucidated. These findings could have a significant impact on interpretations of prior $R_{2}$ and $R_{2}^{*}$ datasets and on future research.

## CONFLICT OF INTEREST STATEMENT

The author has no conflicts of interest to declare.
